# Human Activity Recognition Using Cascaded Dual Attention CNN and Bi-Directional GRU Framework

**DOI:** 10.3390/jimaging9070130

**Published:** 2023-06-26

**Authors:** Hayat Ullah, Arslan Munir

**Affiliations:** Department of Computer Science, Kansas State University, Manhattan, KS 66506, USA; hayatu@ksu.edu

**Keywords:** convolutional neural network, channel–spatial attention, activity recognition, gated recurrent unit, pattern recognition, deep learning

## Abstract

Vision-based human activity recognition (HAR) has emerged as one of the essential research areas in video analytics. Over the last decade, numerous advanced deep learning algorithms have been introduced to recognize complex human actions from video streams. These deep learning algorithms have shown impressive performance for the video analytics task. However, these newly introduced methods either exclusively focus on model performance or the effectiveness of these models in terms of computational efficiency, resulting in a biased trade-off between robustness and computational efficiency in their proposed methods to deal with challenging HAR problem. To enhance both the accuracy and computational efficiency, this paper presents a computationally efficient yet generic spatial–temporal cascaded framework that exploits the deep discriminative spatial and temporal features for HAR. For efficient representation of human actions, we propose an efficient dual attentional convolutional neural network (DA-CNN) architecture that leverages a unified channel–spatial attention mechanism to extract human-centric salient features in video frames. The dual channel–spatial attention layers together with the convolutional layers learn to be more selective in the spatial receptive fields having objects within the feature maps. The extracted discriminative salient features are then forwarded to a stacked bi-directional gated recurrent unit (Bi-GRU) for long-term temporal modeling and recognition of human actions using both forward and backward pass gradient learning. Extensive experiments are conducted on three publicly available human action datasets, where the obtained results verify the effectiveness of our proposed framework (DA-CNN+Bi-GRU) over the state-of-the-art methods in terms of model accuracy and inference runtime across each dataset. Experimental results show that the DA-CNN+Bi-GRU framework attains an improvement in execution time up to 167× in terms of frames per second as compared to most of the contemporary action-recognition methods.

## 1. Introduction

The recent advancements in artificial intelligence (AI), in particular, deep learning-driven vision algorithms, and microelectronics have made possible automated surveillance on Internet of things (IoT) and edge devices [1]. Generally, these surveillance systems are comprised of multiple interconnected cameras deployed in public places, such as offices, roads, shopping malls, hospitals, and airports, to enhance public safety and security [2]. The primary objective behind the deployment of surveillance systems in the aforementioned places is to instantly detect abnormalities by recognizing the anomalous human behavior or activity in a video stream which could result in injury or unlawful conduct. Human activity recognition is a process to analyze the hidden sequential pattern and predict the status of activity based on the perceptual context in input video streams. Generally, in videos, human activity is a combination of different movements of human body parts (i.e., hands, legs, or a combination of both). For instance, running involves rapid movement of hands and legs; similarly, throwing object involves the backward and forward force of arms and hands. Human activity recognition has numerous potential applications, such as in smart surveillance systems [3], video summarization [4], content-based video retrieval [5], sports and healthcare [6], and human–computer interactions [7]. In video, each frame contributes spatial information in sequential order which forms a sequential pattern containing human activity that cannot be recognized in a single video frame. Throwing a ball or a dart (which involves forward and backward force of an arm and hand) have the same action pose in the starting frame and thus the discrimination between these two distinct activities is challenging with respect to recognizing the action in a single frame. Investigating the same movements of an arm and hand in succeeding frames together with the information from previous frames will enable effective recognition of human activities in video stream data.

The earlier developed methods in initial research for vision-based activity recognition are exclusively focused on activities performed by a single person/actor in simple and controlled environments. In contrast, the current research focuses on more challenging and realistic human activities recorded with a cluttered complex background, variation in viewpoint, occlusion in background, inter- and intra-class variations, and pose variations. The existing vision-based human activity-recognition methods can be categorized into two classes namely: (i) traditional handcrafted feature-based, and (ii) deep learning-based human activity-recognition methods. The traditional handcrafted feature-based methods [8,9,10,11,12] use manually designed handcrafted or hand-engineered features (which requires extensive human efforts with prior knowledge of scene understanding) followed by statistical machine learning models to recognize the activity. For instance, several traditional image features have been utilized to analyze videos, such as histogram of 3D oriented gradients (HOG3D), histogram optical flow (HOF) [13], motion boundary histogram (MBH) [14], and extended speeded up robust feature (SURF) feature descriptors. The hand-engineered features must be designed specifically for each particular environment based on scene perceptual complexity. These types of manually designed handcrafted feature-based methods are ineffective while tackling long-term temporal dependencies and complex scenes. Recently, deep learning-based methods have made incredible breakthroughs in various domains of image processing and computer vision, and have been actively used for human activity recognition [15,16,17,18,19]. These deep learning-based methods have obtained state-of-the-art performance by extracting deep progressive discriminative features using different convolutional neural network (CNN) kernels and exploiting a gradient learning strategy. Unlike traditional handcrafted features of an image, deep CNNs learn progressively strong features (containing low-level, mid-level, and high-level features) that help to keep track of all types of visual semantics in image data.

Deep learning-based methods have enhanced the activity-recognition solutions in two perspectives. First, CNNs have the ability to extract more generic and semantically rich features than those of traditional handcrafted feature descriptors. Due to this generic feature extraction enabled by CNNs, CNNs have proliferated in a variety of complex computer vision tasks including 3D image reconstruction [20], image and video captioning [21], and text-to-image generation [22] that cannot be accomplished using traditional handcrafted feature-based methods due to their limitations in terms of features and learning strategies. Secondly, deep learning offers efficient architectures called recurrent neural networks (RNNs) which have the ability to learn representations of human activity from a batch of frames (sequence of frames or temporal representation of human activity) rather than a single frame. Earlier traditional methods consider frame-level classification of human activity in videos, rather than understanding the activity in a sequence of frames that greatly limits their performance for complex and multi-person activities. To cope with this challenge, deep learning-based methods have adopted RNNs for better understanding and recognition of complex human activities in videos. Normally, in deep learning-based methods, RNNs are placed right after CNNs, where the CNN architecture is responsible for extracting deep discriminative features from videos and the RNN is responsible for learning the hidden sequential patterns in the extracted CNN features. The performance of these deep learning methods is good compared to traditional methods; however, these methods are computationally very expensive due to their hybrid and complex CNN and RNN architectures.

The above-mentioned deep learning-based activity-recognition methods have attained exceptional performance. Most of the existing AI-assisted activity-recognition methods have adopted large yet effective pre-trained CNN architectures trained on a large-scale image dataset having tens of millions of trained parameters. Fusing such a computationally expensive feature descriptor backbone architecture with long short-term memory (LSTM) networks or multi-layer LSTMs (LSTMs having several layers with the same settings) greatly increases the computational complexity of the overall method, thereby compromising on the better tradeoff between model accuracy and complexity. Considering the demand for computationally efficient yet effective approaches that provide a balanced tradeoff between model accuracy and complexity for deployment on resource-constrained IoT and/or edge devices, in this paper, we propose a deep learning-based computationally efficient yet effective method for activity-recognition problems that can be deployed even on resource-constrained edge devices in the IoT-enabled surveillance environment. Our main contributions in this work are as follows:

1. We propose a computationally efficient cascaded spatial–temporal learning approach for human activity recognition. The proposed system utilizes deep discriminative RGB features guided by a channel–spatial attention mechanism and long-term modeling of action-centric features for reliable recognition of human activities in video streams.
2. We propose a light-weight CNN architecture having a total of eight convolutional layers where the maximum number of kernels used per layer is 64 with spatial dimensions 3×3. With these constrained settings, we have developed a compact yet efficient CNN architecture for deep discriminative feature extraction as opposed to complex deep CNNs utilized by other contemporary works in their activity-recognition models using transfer learning.
3. We design a stacked dual channel–spatial attention mechanism with residual skip connection for spatial saliency extraction from video frames. The developed dual attentional module is placed after each two-consecutive convolutional layers of the developed CNN model which helps the network to extract saliency-aware deep discriminative features for localizing the action-specific regions in video frames.
4. We propose a bi-directional GRU network with three bi-directional layers (having forward and backward pass) that efficiently capture the long-term temporal patterns of human actions in both forward and backward directions, which greatly enhances the reusability of features, improves the feature propagation, and alleviates the issue of gradients vanishing.
5. We demonstrate the effectiveness and suitability of the proposed encapsulated dual attention CNN and bi-directional GRU framework (DA-CNN+Bi-GRU) for resource-constrained IoT and edge devices by comparing the model accuracy and execution/inference time of the DA-CNN+Bi-GRU framework with various baseline methods as well as contemporary human action-recognition methods.

The remainder of this paper is organized as follows. Section 2 provides a brief overview of the related works covering different types of methods introduced for human activity recognition. The proposed DA-CNN+Bi-GRU framework and its technical components are discussed in detail in Section 3. Section 4 presents an extensive experimental evaluation of DA-CNN+Bi-GRU as compared to other methods based on different metrics. Finally, Section 5 concludes the paper with possible future research directions.

## 2. Related Works on Human Activity Recognition

In recent years, human action and activity recognition have been widely studied and have received an exceptional amount of attention from computer vision researchers due to the recent success of deep learning for image-classification and object-detection tasks. Comprehensive reviews of both traditional and deep learning-based methods have been presented in numerous surveys [23,24]. The reported literature on human action and activity recognition can be summarized in terms of handcrafted feature-based methods, deep learning feature-based methods, long-term temporal modeling-based methods, and attention model-based methods. This section presents a brief discussion on these representative methods and a brief summary of previous related works.

### 2.1. Handcrafted Feature-Based Methods

Numerous traditional handcrafted feature-based methods have been proposed to localize spatial and temporal variations in videos using manually hand-engineered feature descriptors. Generally, these handcrafted feature-based methods can be structured as a feature-extraction and -encoding pipeline having three phases including key feature point detection (spatial and temporal feature points), quantization of detected features, and feature encoding. The first phase involves the extraction of spatial–temporal features from video frames, followed by feature quantization in the second phase which quantizes local motion-centric features. Lastly, the quantized spatial–temporal features are then encoded into feature vectors (known as action feature vectors) having fixed dimensions. For instance, inspired by the feature-extraction mechanism of the scale-invariant feature transform (SIFT) descriptor, Scovanner et al. [25] adopted the SIFT algorithm feature-extraction strategy and extended their feature space from 2D to 3D for encoding hidden action patterns. As single feature representation is not able to capture human actions, numerous multi-feature representative descriptors have been proposed in the literature. Laptev et al. [26] have proposed a multiscale spatial–temporal feature-based approach by utilizing space–time extension and the Harris operator. They first extract multi-scale spatial–temporal features from video frames and then characterize the appearance and motion of local features using a volumetric histogram of oriented gradients (HOG). The retrieved multi-scale spatial–temporal features are then fed to a non-linear support vector machine (SVM) for action recognition. In [27], Ryoo and Matthies inspected the behavior of local and global motion features to recognize first person activities in video data. Their proposed methods exclusively focus on temporal structures depicted in first person action/activity videos. These traditional handcrafted feature-based methods have shown progressive improvement over the years by presenting more efficient approaches; however, these methods are time-consuming (lacking end-to-end recognition strategy), labor-intensive (requiring extensive human efforts to extract generic and more discriminative features), and difficult to adopt in diverse scenarios.

### 2.2. Deep Learning Feature-Based Methods

Deep learning feature-based methods are the current mainstream methods to solve the problem of complex human action and activity recognition in videos. With the recent success in the computer vision domain for high-level vision tasks including image enhancement [28], image segmentation [29], and video captioning [30], CNNs have been actively investigated, addressing human action and activity-recognition problems. Numerous CNN-assisted methods have been presented [31,32,33,34,35,36,37] with deep CNN architectures with 2D convolution kernels applied across convolutional layers of the CNN. These convolutional layers extract deep discriminative spatial features with translation invariance from action video frames, offering reasonable action-recognition performance without using temporal modeling. For instance, Karpathy et al. [31] presented a single-stage CNN architecture for action recognition where they trained their proposed model on a large-scale sports video dataset benchmark, namely the Sports-1M dataset. Although their method achieves better results than traditional handcrafted feature-based methods, the presented architecture is unable to cope with temporal modeling. To overcome this issue, several two-stream CNN architectures were introduced [32,33,37] to obtain both spatial and temporal modeling of human action where one architecture performs spatial modeling of spatial contextual features and the second architecture performs temporal modeling using extracted optical flow features. The addition of a second network improves the performance by introducing temporal modeling to the CNN-based action-recognition approach; however, it equally increases the computational complexity of the overall two-stream CNN approach. To achieve spatial modeling and temporal cues within a single CNN architecture without compromising on model complexity, 3D CNNs [38,39,40] were introduced for human action-recognition tasks. For instance, Tran et al. [38] exploit the powerful characteristics of 3D CNN to recognize human action in sports videos, where they trained their proposed architecture on a large-scale benchmark dataset and have shown promising results. However, these 3D CNN-based approaches work well with short-term temporal modeling and lack the ability to cope with long temporal modeling. Oikonomou et al. [41] examined the effectiveness of advanced data-driven classifiers for action recognition by focusing on a specific set of joint coordinates (x, y, and z-axes). The underlying assumption is that observing this particular set of joints is sufficient for accurately perceiving each action in real-life scenarios. Their objective is to explore the capabilities of joint analysis in improving pose-based action-recognition systems. Consequently, they established correlations between specific joints and corresponding actions, identifying the most influential joints in the process. Shah et al. [42] addressed the task of action recognition using joint-based information. In contrast to alternative modalities, they leveraged the arrangement of joints and their motion to create concise models that capture essential human motion details for activity recognition. Their approach introduces a novel model for joint-based action recognition. The model initially extracts motion features from individual joints independently using a shared motion encoder, and subsequently performs collective reasoning. Holte et al. [43] examined the latest methods in multi-view techniques for the estimation of 3D human poses and recognition of human activities. They explored various fields where these technologies are applied and the specific needs associated with them. These areas include advanced human–computer interaction (HCI), assisted living, interactive games based on gestures, intelligent driver assistance systems, movies, 3D TV and animation, physical therapy, autonomous mental development, smart environments, sport motion analysis, video surveillance, and video annotation. They then conducted a thorough analysis of recent approaches proposed to meet these specific requirements and categorized them accordingly. Nandagopal et al. [44] proposed a novel method for activity recognition called KPE-DCNN. This technique involves several stages: the input video is first transformed into a series of frames, followed by key point extraction using a customized OpenPose model. The extracted key points are then used to classify human activities by training an optimized DCNN model. The goal of KPE-DCNN is to accurately extract key points and effectively recognize different activities based on these points. Zhou et al. [45] proposed a cascaded architecture to tackle the complex task of multi-stage, coarse-to-fine human–object interaction (HOI) understanding. In their approach, each stage of the architecture consists of an instance localization network that progressively refines HOI proposals. These refined proposals are then passed on to an interaction-recognition network. Notably, both networks maintain connections with their respective predecessors from the previous stage, facilitating cross-stage information exchange. The interaction-recognition network comprises two key components: a relation ranking module for selecting high-quality HOI proposals and a triple-stream classifier for relation prediction. These modules synergistically utilize carefully designed human-centric relation features to achieve effective interaction understanding. To study instance-aware posing of human body parts, Zhou et al. [46] proposed a novel bottom-up approach that simultaneously addresses the tasks of category-level human semantic segmentation and multi-person pose estimation in a joint and end-to-end fashion. This approach yields a compact, efficient, and robust framework that leverages structural information across various levels of human granularity, thereby mitigating the challenges associated with person partitioning. The key innovation of this work is the learning of a dense-to-sparse projection field, which facilitates the explicit association of dense human semantics with sparse keypoints. This projection field is progressively refined throughout the network’s feature pyramid, resulting in an improved performance and representation of the relationships between human semantics and pose keypoints. The resulting framework benefits from its ability to incorporate and enhance structural information, providing a powerful tool for the given tasks. 

### 2.3. Temporal Modeling-Based Methods

The temporal modeling approach has been actively used to overcome the issue of long-term temporal modeling, where researchers have introduced a special kind of neural network called RNN, which has the ability to deal with long-term sequences. Different variants of RNNs have been introduced for action-recognition problems including LSTM [47], bi-directional LSTM [48], and GRU [49], which are comparatively more efficient than RNNs in terms of memorizing contents for long periods of time. For instance, Yue et al. [34] presented a two-stream CNN architecture to extract both spatial (edge, color and shape) features and temporal (optical flow) features stacked with the LSTM model for temporal modeling of human activity. Similarly, Amin et al. [47] presented a two-stream CNN architecture followed by a multi-layer LSTM to recognize human activity in videos. They first extracted spatial salient and optical flow features and then fed the extracted features to multi-layer LSTM to localize human action in video sequences. Ibrahim et al. [50] proposed a two-stream temporal modeling-based activity-recognition framework to recognize a team or group of activities. Their proposed method consist of two LSTM networks; the first LSTM learns the representation of a single person action, whereas the second LSTM is responsible for understanding collective activity by aggregating individual actions in a sequence of frames. Biswas et al. [51] presented a special variant of RNN named structural RNN for group activity recognition. Their proposed method consists of series of interconnected RNNs structured to analyze human actions and their mutual interactions in video sequences. To accurately learn representations of human activity in feature-encoded video frames, Shugao et al. [52] reformulated ranking loss to efficiently detect human activities. They first extracted deep discriminative CNN features from video frames using VGG19, which are then fed into LSTM to analyze hidden sequential patterns and recognize human activities. Muhammad et al. [6] presented a spatio-termporal approach for recognizing salient events in soccer videos, where they used a pretrained ResNet50 architecture for deep feature extraction and a multilayer LSTM for event recognition from the hidden sequential patterns. These hybrid CNN+LSTM approaches exhibited significant performance for vision-based human action- and activity-recognition tasks; these methods are computationally complex due to intensive computation caused by CNN feature extraction and human action modeling by LSTM.

### 2.4. Attention Mechanism-Based Methods

In the recent past, attention-based method have demonstrated great potential for a variety of high-level vision tasks including image segmentation [53], video captioning [54], and visual question answering (VQA) [55]. More recently, attention mechanisms combined with CNN and RNN networks have been widely used for human action-recognition tasks and have achieved noticeable improvements in action-recognition performance. For instance, Baradel et al. [56] introduced a novel spatio-temporal attention mechanism for human action recognition. Their approach automatically directs attention to the most significant human hands and detects the most discriminative moments within an action. Unlike conventional soft-attention mechanisms, they employed an RNN to handle attention in a fully differentiable manner. Notably, they diverged from the typical practice of using the hidden RNN state as an input to the attention model. Instead, they generated attention distributions using external information, specifically human articulated poses. Islam et al. [57] proposed Multi-GAT, a hierarchical multi-modal HAR approach that incorporates graphical attention. Their method focuses on learning complementary features from multiple modalities in a hierarchical manner. To disentangle and extract salient modality-specific features facilitating feature interactions, they devised a multi-modal mixture-of-experts model. Furthermore, they introduced a novel message-passing-based graphical attention approach, which captures cross-modal relations to extract complementary multi-modal features. Long et al. [58] introduced *keyless attention* as a sophisticated and efficient approach to better address the sequential characteristics of data. Additionally, through a comprehensive comparison of various multi-modal fusion techniques, they discovered that multimodal keyless attention fusion achieves the highest success in capturing interactions between different modalities. Song et al. [59] proposed a spatial and temporal attention model to investigate discriminative features in human action recognition and detection using skeleton data. Their approach utilizes RNNs with LSTM units to build the network architecture. Their proposed model learns to selectively focus on discriminative joints within each input frame and assigns varying levels of attention to the outputs of different frames. To ensure effective training for action recognition, they introduced a regularized cross-entropy loss and devised a joint training strategy. Additionally, leveraging temporal attention, they developed a technique to generate temporal proposals for action detection. Cho et al. [60] introduced three variations of the self-attention network (SAN) called SAN-V1, SAN-V2, and SAN-V3. These variants effectively extract high-level semantics by capturing long-range correlations. The authors also incorporated a temporal segment network (TSN) into their SAN variants, leading to notable enhancements in overall performance. Although these attention-driven methods have been widely used for human action-recognition task and have obtained noticeable improvements over handcrafted feature-based methods and other non-attention deep learning methods, they perform well only on clean red, green, and blue (RGB) video data and mostly fail while dealing with noisy color (RGB) video data.

## 3. Proposed Human Activity-Recognition Framework

**Figure 1:**
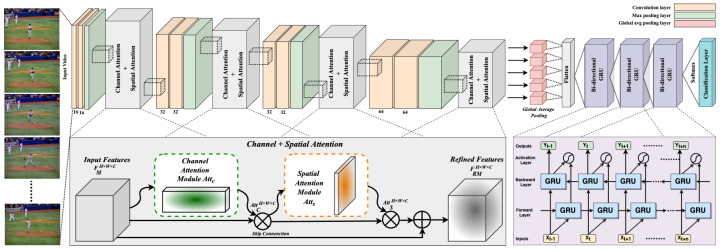
A graphical overview of our proposed activity-recognition framework. The proposed framework consists of three main modules: CNN architecture, dual channel and spatial attention module, and bi-directional GRU network. The CNN module utilizes a dual-attention mechanism to effectively extract salient CNN features from video frames, whereas the bi-directional GRU network is used to learn the activity representation for hidden sequential patterns.

This section presents in detail the insights of our proposed DA-CNN+Bi-GRU human action-recognition framework and its core components. For better understanding, the proposed approach is divided into three distinct modules, where each module is separately discussed. The first core component of our method is the newly introduced lightweight CNN architecture having a small number of trainable parameters. The second core component is a dual attention (channel and spatial attention) module, which is used to embed a dual attention mechanism to the CNN module to enable our CNN model to extract salient features from video frames. The last key component of our framework is a bi-directional GRU network for learning long-term encoded patterns of human actions. The conceptual workflow of the DA-CNN+Bi-GRU framework is depicted in Figure 1.

### 3.1. Overview of Proposed CNN Architecture

Recognizing human actions in video data is indeed a challenging problem, where video data represent complex human actions over a series of frames in the form of different hidden visual contents that include temporal flow of objects in frames, varying textures, object-specific edges, and colors. For better representation and modeling of human actions, these visual contents need to be analyzed effectively via an approach that allows an activity-recognition system to recognize complex human actions or activity in video sequences. To effectively extract the defining visual features of these hidden action contents, CNN-based approaches are widely used to recognize human actions in videos. Although the presented CNN-based approaches have shown remarkable performance, their computational complexity and execution/inference times are very high due to large network architectures. To avoid such high computational complexity and long runtime, we propose a lightweight CNN architecture coupled with channel and spatial attention. The proposed CNN architecture contains a total of eight convolutional layers, where each two consecutive convolutional layers are followed by a max pooling layer and a dual attention block (containing both channel and spatial attention). The first two convolutional layers each apply 16 kernels on input video frames with the kernel size 3×3, whereas the third and fourth convolutional layers each apply 32 kernels on the output of the first dual attention block with the kernel size 3×3.

Similarly, the fifth and sixth convolutional layers each apply 32 kernels on the output of the second dual attention block with the kernel size 3×3. The last pair of the convolutional layers each apply 64 kernels on the output of the third dual attention block with the kernel size 3×3 and then forward the estimated feature maps to the last dual attention block. The output of the last dual attention block is processed by a global average pooling layer, the output of which is then flattened by a flatten layer. The output of the flatten layer is fused with a bi-directional GRU network for later long short-term sequence learning. The architectural details of our proposed CNN architecture are listed in Table 1. It is worth noticing that we used at most 64 convolutional kernels per layer and a fixed 3×3 kernel size that greatly help to reduce the computational complexity as low as possible with a negligible effect on model performance.

### 3.2. Dual Attention Module

**Figure 2:**
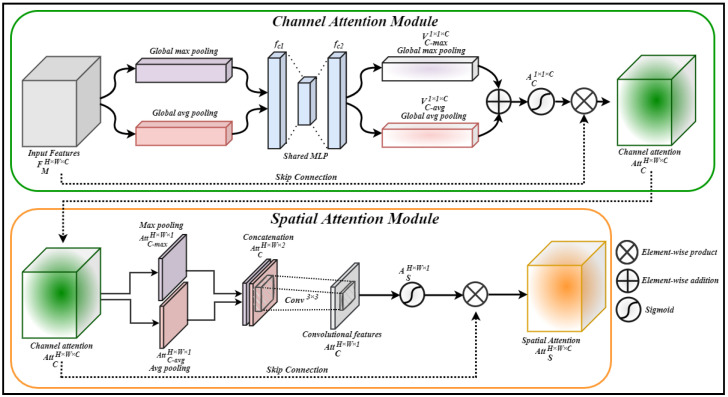
The building blocks of dual attention blocks containing channel and spatial attention mechanisms in detail.

To exclusively focus on the most salient regions of video frames, we propose an attention-driven CNN architecture to efficiently localize the salient regions and enhance feature representation. The proposed attention mechanism is formed by modifying the convolutional block attention module (CBAM) [61] by replacing the 7×7 convolution layer with a 3×3 convolution layer, followed by the fusion of the spatial attention module with the intermediate output of the channel attention module through element-wise product operation. A detailed graphical overview of the proposed dual attention block is presented in Figure 2. The fusion of both channel and spatial attentions not only helps to reduce the overall parameters overhead, but also enables the proposed CNN architecture to extract salient features. Therefore, the formation of network layers is constructed in such a way that we place a stacked dual attention module after each two consecutive convolutional layers of our proposed network. The channel attention module estimates the weighted contribution of RGB channels by applying intermediate channel attention $A_{C}$ on the output feature maps $F_{M}$ of the previous convolutional layer to obtain the channel attention $Att_{C}$. The computed output from $Att_{C}$ is then forwarded to the spatial attention module, which localizes promising object-specific regions by applying spatial attention on the computed channel attention feature maps $Att_{C}$. Finally, the architecture obtains the refined feature maps $F_{RM}$ by fusing the spatial attention feature maps $Att_{S}$ with the input feature maps $F_{M}$ via a residual skip connection using element-wise addition operation. Mathematically, $Att_{C}$, $Att_{S}$, and $F_{RM}$ can be formulated as follows:

(1) $$Att_{C}^{H\times W\times C}=A_{C}\left(F_{M}^{H\times W\times C}\right)⊗F_{M}^{H\times W\times C},$$

(2) $$Att_{S}^{H\times W\times C}=A_{S}\left(Att_{C}^{H\times W\times C}\right)⊗Att_{C}^{H\times W\times C},$$

(3) $$F_{RM}^{H\times W\times C}=Att_{S}^{H\times W\times C}⊕F_{M}^{H\times W\times C}$$

Here, *H*, *W*, and *C* denote the height, width, and number of channels of the feature maps, respectively. $A_{C}$ and $A_{S}$ are the intermediate channel attention and the intermediate spatial attention, respectively. $F_{RM}$ is the final refined feature maps obtained by fusing spatial attention and input feature maps $F_{M}$.

#### 3.2.1. Channel Attention

In pattern-recognition problems, particularly in image/object recognition, each color channel contributes differently based on the appearance of color in an image. During the training process, a CNN model generates feature maps from input image data by extracting deep discriminative features through multiple convolutional layers. Within these feature maps, certain channels have a higher contribution than others in the recognition process, emphasizing their importance in capturing relevant information. Unlike the earlier attention-based approaches that used either global max pooling layer or global average pooling layer, DA-CNN+Bi-GRU uses both global max pooling and global average pooling to extract more effective features. Global max pooling emphasizes highly activated values by selecting the maximum value from the receptive field, whereas global average pooling estimates the equally weighted feature maps for each channel.

The computed feature maps are then forwarded to a shared multilayer perceptron (MLP) containing two fully connected layers, namely fc1 and fc2, having 128 and 512 nodes, respectively. The shared MLP learns the non-linearity between the two fully connected layers using the rectified linear unit (ReLU) activation function, and outputs two individual feature vectors, namely $V_{C-max}^{1\times 1\times C}$ and $V_{C-avg}^{1\times 1\times C}$, for global max pooling and global average pooling, respectively. The computed feature vectors are then combined via an element-wise addition operation, and then forwarded to a sigmoid activation function σ, which normalizes the feature values to obtain intermediate channel attention features $A_{C}^{1\times 1\times C}$. The obtained intermediate channel attention features $A_{C}^{1\times 1\times C}$ are then fused with the input feature maps $F_{M}^{H\times W\times C}$ using a residual skip connection by performing an element-wise multiplication operation, which results in the ultimate channel attention feature maps $Att_{C}^{H\times W\times C}$ as depicted in Figure 2. Mathematically, the channel attention and its components can be expressed as follows:

(4) $$V_{C-max}^{1\times 1\times C}=fc2\left(R_{eLU}\left(fc1\left(maxpool\left(F_{M}^{H\times W\times C}\right)\right)\right)\right),$$

(5) $$V_{C-avg}^{1\times 1\times C}=fc2\left(R_{eLU}\left(fc1\left(avgpool\left(F_{M}^{H\times W\times C}\right)\right)\right)\right),$$

(6) $$A_{C}^{1\times 1\times C}=\sigma \left(V_{C-max}^{1\times 1\times C}⊕V_{C-avg}^{1\times 1\times C}\right),$$

(7) $$Att_{C}^{H\times W\times C}=A_{C}^{1\times 1\times C}⊗F_{M}^{H\times W\times C},$$

Here, $V_{C-max}^{1\times 1\times C}$ and $V_{C-avg}^{1\times 1\times C}$ are the feature vectors obtained from global max pooling and global average pooling operations, respectively. In the above equations, $F_{M}^{H\times W\times C}$ represents the input feature maps, σ denotes the sigmoid activation function, whereas $Att_{C}^{H\times W\times C}$ is the final channel attention output.

#### 3.2.2. Spatial Attention

The spatial attention mechanism focuses on object saliency in the given feature maps by paying more attention to important features across each color channel and localizing salient regions. To highlight the salient object-specific regions in the feature maps, our design exploits inter-spatial features and their relationship among channels, which greatly help to trace the target object in the feature maps. DA-CNN+Bi-GRU computes the relation of inter-spatial features among channels by applying max pooling and average pooling to the input channel attention feature maps to obtain max pooled channel attention $Att_{C-max}^{H\times W\times 1}$ and average pooled channel attention $Att_{C-avg}^{H\times W\times 1}$, respectively.

**Figure 3:**
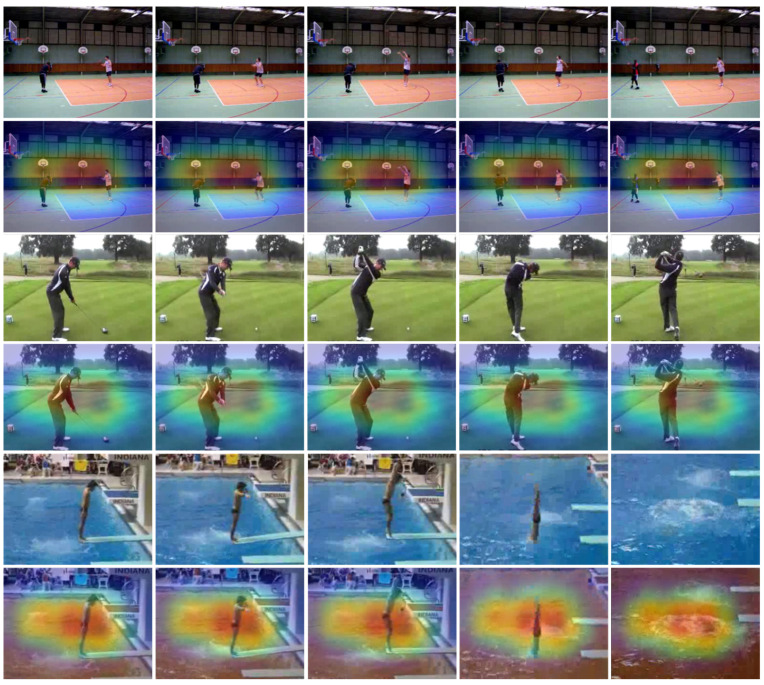
Visual representation of the salient object-specific regions computed with our dual attention mechanism.

The max pooled channel attention $Att_{C-max}^{H\times W\times 1}$ and average pooled channel attention $Att_{C-avg}^{H\times W\times 1}$ are concatenated and then forwarded to a single convolutional layer $Conv^{3\times 3}$, which applies a 3×3 convolution kernel on pooled feature maps to form single-channel convoluted feature maps. These convoluted feature maps are then processed by a sigmoid activation function, which normalizes the learned features and produces intermediate spatial attention features $A_{S}^{H\times W\times 1}$. Finally, the obtained intermediate spatial attention features $A_{S}^{H\times W\times 1}$ are fused with the input channel attention feature maps $Att_{C}^{H\times W\times C}$ using a residual skip connection by performing an element-wise multiplication operation, which results in final spatial attention feature maps $Att_{S}^{H\times W\times C}$, as depicted in Figure 2. Mathematically, spatial attention $Att_{S}^{W\times H\times C}$ and its component can be expressed as follows:

(8) $$Att_{C-max}^{H\times W\times 1}=maxpool\left(Att_{C}^{H\times W\times C}\right),$$

(9) $$Att_{C-avg}^{H\times W\times 1}=avgpool\left(Att_{C}^{H\times W\times C}\right),$$

(10) $$A_{S}^{H\times W\times 1}=\sigma \left(Conv^{3\times 3}\left(Att_{C-max}^{H\times W\times 1}⊎Att_{C-avg}^{H\times W\times 1}\right)\right),$$

(11) $$Att_{S}^{H\times W\times C}=A_{S}^{H\times W\times 1}⊗Att_{C}^{H\times W\times C},$$

where $Att_{C-max}^{H\times W\times 1}$ and $Att_{C-avg}^{H\times W\times 1}$ are the global max and average pooled features, respectively. σ is the sigmoid activation function and ⊎ represents the concatenation operation. $Att_{S}^{H\times W\times C}$ is the final obtained spatial attention. The representative saliency maps of different human actions generated by our proposed method are depicted in Figure 3.

### 3.3. Learning Human Action Patterns via Bi-Directional GRU

Videos can be conceptualized as a stack of frames that encapsulate the sequential flow of diverse visual contents within a specific time duration. To understand the visual contents, mainstream computer vision approaches first extract deep discriminative features from the video frames using CNNs and then combine the extracted features in sequential order to maintain the semantic flow of the video. Second, the feature-encoded videos are then processed via RNNs to learn the representation of visual contents from hidden sequential patterns. Specifically, for human activity-recognition problems, two special variants of RNNs are actively used by researchers that include LSTMs and GRUs. The LSTM unit is comprised of different gates including input, output, forget gates, and other memory components, whereas the GRU contains an update gate, a reset gate, and an activation function. The LSTM is comparatively more complex than the GRU in terms of the number and formation of gates which leads to relatively higher computational complexity requiring more computational resources. Therefore, in this paper, we propose to use GRU with bi-directional flow of learning strategy, which effectively learns from the encoded hidden sequential pattern.

**Figure 4:**
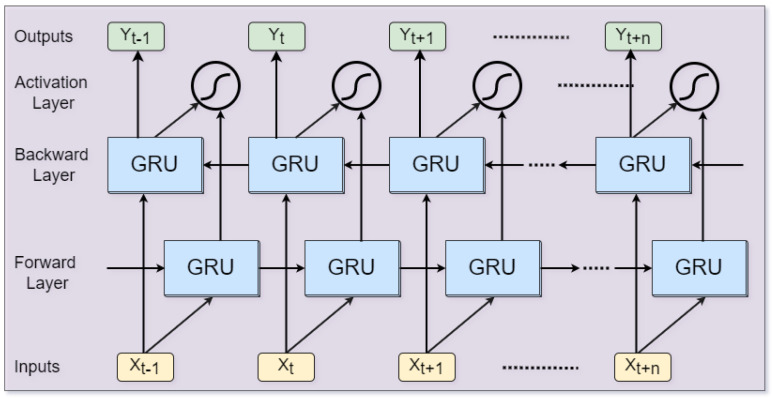
The building block of bi-directional single GRU layer.

The bi-directional GRU consists of two layers, namely forward and backward layer, where both layers process the same sequence in different sequential order. The forward layer reads the input sequence from left to right, that is, from $X_{t-1}$ to $X_{n}$ where *n* is the length of sequence. On the other hand, the backward layer reads the input sequence in reverse order from right to left, that is, from $X_{t+n}$ to $X_{t-1}$ as shown in Figure 4. Both forward and backward GRU layers consist of GRU cells, where each cell consists of two gates, namely a reset *r* and an update gate μ, with two activation functions that include sigmoid and tanh. The reset gate decides whether the GRU needs to forget or retain the portion of information based on its values (between 0 and 1). When the output value of the reset gate is near 0, the reset gate forgets the information from the previous portion of the sequence, whereas if the reset gate value is near 1, the reset gate retains the previous portion of the sequence. The update gate decides the amount of information from the previous hidden state to be retained to the current hidden state based on its values (between 0 and 1). When the value of the update gate is near 0, the updated gate simply forgets the portion of information from the previous hidden state and retains the portion of information from the previous hidden state to the current hidden state when the value is close to 1. Mathematically, the operation of these gates can be expressed as follows:

(12) $$r_{t}=\sigma \left(w_{r}\cdot x_{t}+u_{r}\cdot h_{t-1}\right),$$

(13) $$\mu_{t}=\sigma \left(w_{\mu }\cdot x_{t}+u_{\mu }\cdot h_{t-1}\right),$$

(14) $${\tilde{h}}_{t}=\tanh\left(w\cdot x_{t}+r_{t}\cdot u\cdot h_{t-1}\right),$$

(15) $$h_{t}=\left(1-\mu_{t}\right)\cdot h_{t-1}+\mu_{t}\cdot {\tilde{h}}_{t},$$

(16) $$y_{t}=\sigma \left(w_{o}\cdot h_{t}\right),$$

where $r_{t}$ and $\mu_{t}$ represent the reset and update gates, respectively, having values between 0 and 1. In the above equations, *w* and *u* are the weight variables, $x_{t}$ is the input to the GRU layer, $w_{o}$ is the weight variable between input and output layer, $y_{t}$ represents the output layer node at time step t. ${\tilde{h}}_{t}$ is the candidate hidden state of the current node, $h_{t}$ is the current hidden state, and $h_{t-1}$ is the hidden state of the previous node.

## 4. Experimental Results and Discussion

In this section, we present a detailed experimental evaluation of our proposed DA-CNN+Bi-GRU human activity-recognition framework. We evaluate the effectiveness of our proposed framework by analyzing the performance with and without the key components (channel attention, spatial attention, bi-directional GRU) of our framework. First, we describe the implementation details and performance evaluation metrics that we used in this research. Next, we briefly discuss the datasets we used for benchmarking experiments. We then compare the DA-CNN+Bi-GRU framework with state-of-the-art human action-recognition methods across each tested dataset. Finally, we present the human action-recognition visualization and then conduct runtime analysis of our proposed approach for real-time human activity recognition.

### 4.1. Implementation Details

The DA-CNN+Bi-GRU framework is implemented using a well-known deep learning framework called TensorFlow version 2.0 in Python language 3 on a computing system with an Intel Xeon (R) processor with processor frequency 3.50 GHz and 32 GB of dedicated main memory. The computing system is also equipped with an NVIDIA GeForce GTX 1080 graphics processing unit (GPU) having a graphics random-access memory of 8 GB. For training and validation, we divided the datasets into a ratio of 70% and 30%, where for training we used 70% of the data and the remaining 30% of the data were used for validation. The training process is run for 300 epochs and the weights are initialized with a random weight initializer, whereas the batch size is set to 16. To adjust weight values during training, we used the Adam optimizer with static learning rate of 0.0001. The DA-CNN+Bi-GRU network utilizes categorical cross-entropy loss, which controls the weight adjustment based on network prediction during training. For sequence learning, we used a sequence length of 16 frames without overlapping for both forward and backward pass of bi-directional GRU, where we used three bi-directional GRU layers with 32 GRUs per layer. Moreover, we used two different performance evaluation metrics to assess the overall performance of our proposed method. The first metric is the *accuracy* metric, which is used to evaluate the activity-recognition performance of our framework and other contemporary methods. The second metric is *frames per second (FPS)* or alternatively *seconds per frame (SFP)*, which measures the runtime of our proposed framework and other contemporary methods.

### 4.2. Datasets

To verify the effectiveness of the DA-CNN+Bi-GRU framework, we conducted extensive experiments on three challenging human action datasets that include YouTube action, UCF50, HMDB51, UCF101, and Kinetics-600 datasets. Each dataset consists of multiple action videos having varying duration, different viewpoints, and FPS. These datasets are discussed in detail in the following subsections.

#### 4.2.1. YouTube Action Dataset

The YouTube action dataset [62] is a commonly used action-recognition dataset containing diverse sports and other action video clips collected from YouTube. The collected videos clips are very challenging due to variations in viewpoint, camera motion, cluttered background, and varying poses and appearances of objects in the scene. The dataset contains 1640 video clips categorized into 11 action categories, where the duration of videos ranges between 2 and 5 s having a frame rate of 29 FPS and a resolution of 320×240. The collected action clips in all action categories are grouped into 25 distinct groups containing four or more video clips, where each video clip in the same group shares common visual features, such as background, viewpoint, and the person or actor.

#### 4.2.2. UCF50 Dataset

The UCF50 dataset [63] is one of the challenging large-scale human activity-recognition datasets, containing videos of diverse human actions captured with varying viewpoints, camera motions, object poses and appearances, and background clutter. The dataset contains a total of 6676 video clips categorized into 50 different classes, where the duration of video clips ranges between 2 and 3 s with a frame rate of 25 FPS and a resolution of 320×240. The video clips in all 50 categories are further grouped into 25 groups, where each group comprises at least four video clips, where a video clip in a single group shares common features of actions, such as the same person performing an action, the same viewpoint, and the same background.

#### 4.2.3. HMDB51 Dataset

HMDB51 [64] is one of the challenging datasets commonly used for human action recognition in videos. The videos in this dataset are collected from difference sources including movies, public databases, YouTube, and Google videos. The dataset comprises a total of 6849 action video clips categorized into 51 classes, where each class contains at least 101 video clips having a duration of 2 to 3 s with a frame rate of 30 FPS and a resolution of 320×240. The collected action video clips can be generally categorized into five different types of actions that include facial actions, facial actions with object manipulation, general body movements, body movements and interaction with objects, and body movements while interacting with humans.

#### 4.2.4. UCF101 Dataset

According to the literature based on human action/activity recognition, UCF101 [65] is a very challenging dataset comprising videos which resemble real-world activities. The dataset consists of 13,320 videos collected from YouTube, which are categorised into 101 action classes, where in each class there are 100 to 200 video clips of human actions performed by different subjects. The duration of each clip is between 2 and 3 s with a frame rate of 25 FPS and a frame resolution of 320×240. The collected video clips are retrieved based on five major human activities that include human interaction with objects, human interaction with humans, human body motion only, playing of musical instruments, and humans performing sports activities.

#### 4.2.5. Kinetics-600 Dataset

Kinetics-600 [66] is an extremely large human action video dataset, comprising 480 K video clips (∼10 s) of different human actions that are categorized into 600 actions classes. The video clips in the dataset are collected from YouTube videos which are then labeled based on the action in video clips, where each video clip has a variable resolution, field of view, and frame rate. The dataset has three distinct sets, namely train, validation, and test. The train set contains 500∼950 videos per class, whereas in the validation set, each class has 45∼50 videos. The test set and validation set have the same number of videos; however, the test set is not labeled.

### 4.3. Assessment of Our Framework with Baseline Methods

This research is built up on the exploration of various possible solutions for vision-based human action recognition, where we developed several spatial–temporal methods, assessed their performances, and developed our final proposed method. To obtain the optimal approach, we explored different spatial–temporal solutions and successively developed four different baseline methods that include CNN+LSTM, CNN+Bi-LSTM, CNN+GRU, and CNN+Bi-GRU, and we analyzed their performances in terms of model precision. To obtain a fair comparison, we trained each baseline method on five different datasets (i.e., YouTube action, UCF50, HMDB51, UCF101, and Kinetics-600 datasets). These datasets are then used for training the DA-CNN+Bi-GRU framework. The network settings of these baseline methods are listed in detail in Table 2, where it can be perceived that CNN+LSTM and CNN+GRU methods use a total of 11 spatial–temporal layers including 8 convolutional and 3 temporal layers. Similarly, CNN+Bi-LSTM and CNN+Bi-GRU methods use a total of 14 layers, including 8 convolutional and 6 temporal layers (with 3 forward and 3 backward pass layers). Finally, the proposed framework (DA-CNN+Bi-GRU) has a total of 18 layers comprising 12 convolutional layers (8 convolutional and 4 attentional) and 6 temporal layers (3 forward and 3 backward pass layers).

**Figure 5:**
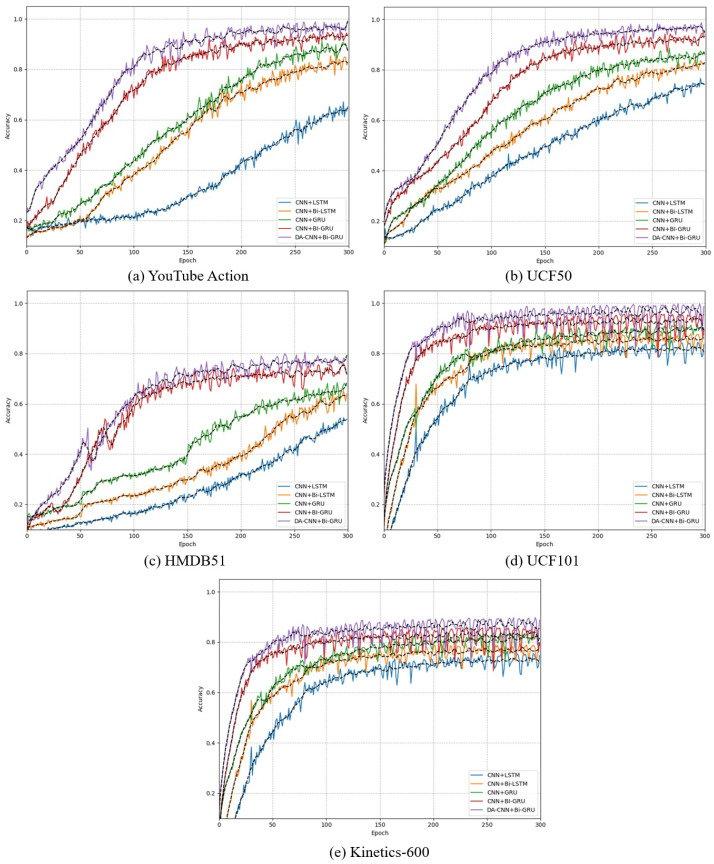
Validation history of our proposed DA-CNN+Bi-GRU framework along with other tested baseline methods for 300 epochs over three benchmark action datasets: (**a**) Validation history for YouTube action dataset, (**b**) Validation history for UCF50 dataset, (**c**) Validation history for HMDB51 dataset, (**d**) Validation history for UCF101 dataset, and (**e**) Validation history for Kinetics-600 dataset.

The training performance (in terms of accuracy) of each baseline method along with our proposed method is depicted in Figure 5. It can be seen from Figure 5 that DA-CNN+Bi-GRU performs better than other baseline methods in terms of accuracy. For instance, in Figure 5a for the YouTube action dataset, our method achieves the best accuracy score throughout 300 epochs. In Figure 5b for the HMDB51 dataset, our method (DA-CNN+Bi-GRU) starts as the second-best method in early training epochs where CNN+Bi-GRU dominates; however, after 20 epochs, DA-CNN+Bi-GRU attains the best accuracy as compared to the other baseline methods and remains the best till the end of the training. Similarly, in Figure 5c for the UCF50 dataset, our method (DA-CNN+Bi-GRU) does not perform the best in the first 35 epochs, where CNN+Bi-GRU dominates; however, after 35 epochs, the DA-CNN+Bi-GRU starts improving and finally trains with the best accuracy at the 300th epoch. In Figure 5d for the UCF101 dataset, the proposed DA-CNN+Bi-GRU starts with the best training accuracy in the very early epochs and shows the best performance throughout the training phase (for 300 epochs) and finishes with the best training accuracy. Finally, in Figure 5e for the Kinetics-600 dataset, the proposed method obtains the best training accuracy throughout the training phase followed by the CNN+Bi-GRU method with the second best performance in terms of training accuracy.

**Figure 6:**
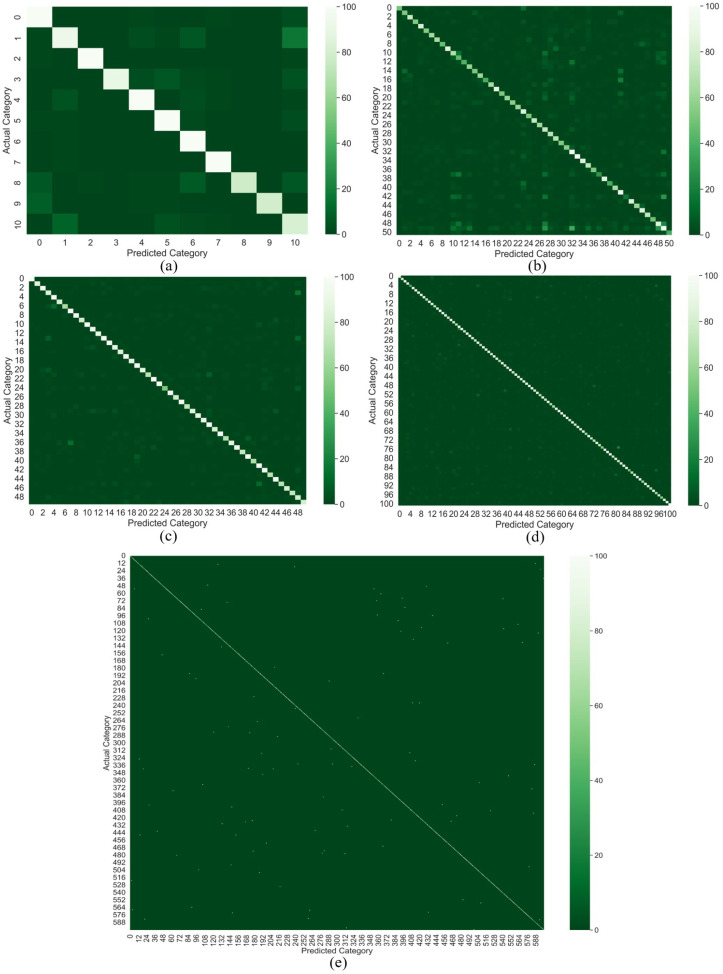
Confusion matrices computed for the proposed DA-CNN+Bi-GRU for the test sets of five tested datasets: (**a**) YouTube Action dataset, (**b**) HMDB51 dataset, (**c**) UCF50 dataset, (**d**) UCF101 dataset, and (**e**) Kinetics-600 dataset.

**Figure 7:**
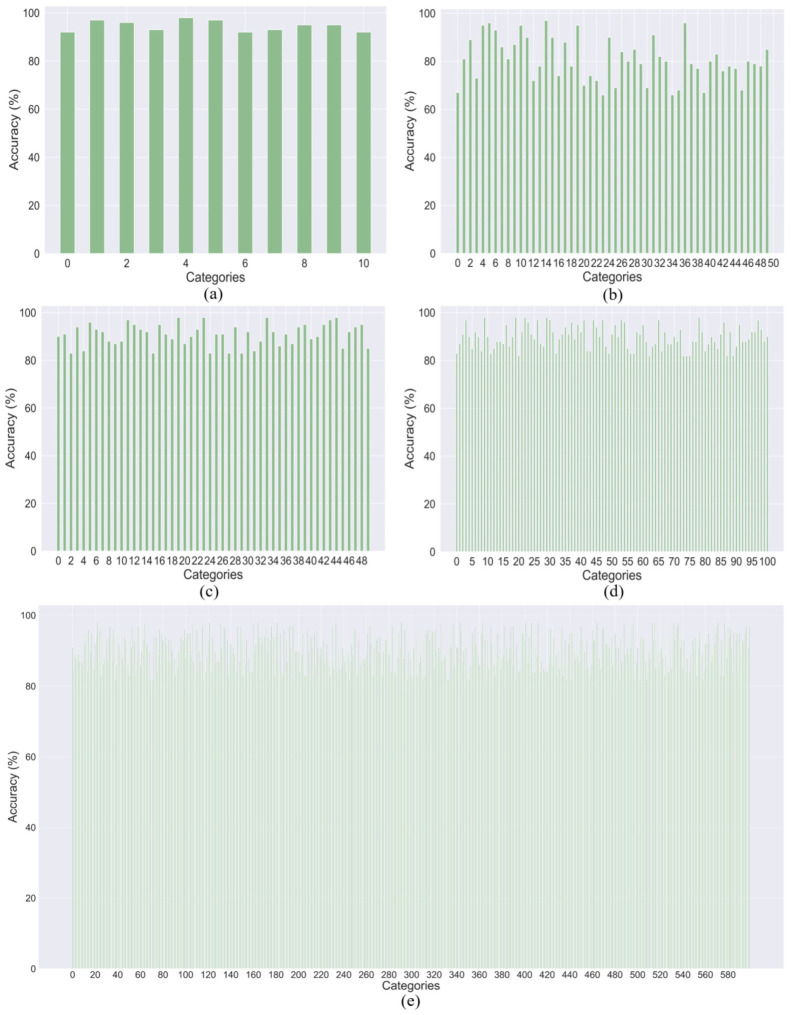
Category-wise accuracy of the proposed DA-CNN+Bi-GRU on the test sets of five tested datasets: (**a**) YouTube Action dataset, (**b**) HMDB51 dataset, (**c**) UCF50 dataset, (**d**) UCF101 dataset, and (**e**) Kinetics-600 dataset.

We also demonstrate the performance of our proposed DA-CNN+Bi-GRU method on YouTube action, HMDB51, UCF50, UCF101, and Kinetics-600 datasets using confusion matrix and category-wise accuracy metrics. The obtained results for the confusion matrix and category-wise accuracy metrics are depicted in Figure 6 and Figure 7, respectively. The obtained performances of these baseline methods along with our proposed method across five benchmark datasets are presented in Table 3. From Table 3, it can be noticed that DA-CNN+Bi-GRU dominates all the baseline methods across each dataset. For instance, the proposed framework attains the best accuracy score of 98.0% over the YouTube action dataset as compared to all the baseline methods, whereas CNN (spatial attention only) + Bi-GRU obtains the second-best accuracy score of 95.6%. Similarly, on the UCF50 dataset, the proposed framework obtains the highest accuracy score of 98.5%, whereas the runner-up is CNN (spatial attention only) + Bi-GRU with an accuracy of 95.7%. For the HMDB51 dataset, it can be seen that our proposed method dominates all the baseline methods by achieving the best accuracy score of 79.3%, whereas CNN (spatial attention only) + Bi-GRU is the runner-up method, attaining the second-best accuracy score of 74.5%. For the HMDB51 dataset, the proposed framework attains the highest accuracy score of 79.3%, whereas CNN (spatial attention only) + Bi-GRU is the runner-up method, obtaining the second-best accuracy of 74.5%. For the UCF101 dataset, the proposed DA-CNN+Bi-GRU outperforms all the baseline methods by obtaining an accuracy of 97.6%, whereas the runner-up is the baseline method CNN (spatial attention only) + Bi-GRU which obtains an accuracy of 95.8%. Finally, for the Kinetics-600 dataset, the proposed DA-CNN+Bi-GRU achieves the best accuracy of 86.7% amongst all the baseline approaches, whereas the CNN (spatial attention only) + Bi-GRU method attains the second best accuracy of 85.6%. The best and the runner-up results are indicated in bold and italics, respectively, in Table 3. 

### 4.4. Comparison with State-of-the-Art Methods

To show the effectiveness of the DA-CNN+Bi-GRU framework for the human activity-recognition task, we conducted an extensive comparative analysis of our method with the state-of-the-art methods in terms of overall accuracy. The quantitative comparisons of our method with the state-of-the-art methods for YouTube action, UCF50, HMDB51, UCF101, and Kinetics-600 datasets are listed in Table 4, Table 5, Table 6, Table 7 and Table 8, respectively. The best results in these tables are represented in bold, whereas the runner-up results are highlighted in italics. Considering the presented results, it can be noticed that DA-CNN+Bi-GRU outperforms state-of-the-art methods on UCF50, HMDB51, and UCF101 datasets, whereas it attains runner-up performance on the YouTube action and Kinetics-600 datasets. For the YouTube action dataset, the STDN [67] has the best performance with an accuracy of 98.2%, whereas DA-CNN+Bi-GRU attains the runner-up performance by obtaining an accuracy of 98.0%, which is within 0.2% accuracy of the best-performing STDN [67]. Thus, for most practical purposes, the DA-CNN+Bi-GRU framework attains performance comparable to STDAN [67]. Other methods compared include multi-task hierarchical clustering [68], BT-LSTM [69], deep autoencoder [70], two-stream attention LSTM [71], weighted entropy-variance-based feature selection [72], dilated CNN+BiLSTM+RB [73], DS-GRU [52], and local-global features + QSVM [74], which obtain 89.7%, 85.3%, 96.2%, 96.9%, 94.5%, 89.0%, 97.1%, and 82.6% accuracies, respectively.

For the UCF50 dataset, DA-CNN+Bi-GRU dominates the state-of-the-art methods by obtaining the best accuracy of 98.5%, whereas (LD-BF) + (LD-DF) [77] obtains the second-best accuracy of 96.7%. Local-global features + QSVM [74] achieves the lowest accuracy of 69.4%, whereas the rest of the methods including multi-task hierarchical clustering [68], deep autoencoder [70], ensemble model with swarm-based optimization [75], DS-GRU [52], and ViT+LSTM [76] obtain 93.2%, 96.4%, 92.2%, 95.2%, and 96.1% accuracies, respectively. For the HMDB51 dataset comprising challenging action videos, our proposed method achieves the best results by obtaining an accuracy of 79.3%, whereas the runner-up method is evidently deep learning [78] which attains an accuracy of 77.0%. The multi-task hierarchical clustering method [68] achieves an accuracy of 51.4%, which is the lowest among all the comparative methods on the HMDB51 dataset. The rest of the comparative methods including STPP+LSTM [79], optical flow + multi-layer LSTM [47], TSN [80], IP-LSTM [81], deep autoencoder [70], TS-LSTM + temporal-inception [82], HATNet [83], correlational CNN+LSTM [84], STDAN [67], DB-LSTM+SSPF [48], DS-GRU [52], TCLC [85], ViT+LSTM [76], semi-supervised temporal gradient learning [86], AdaptFormer [87], SVT (Linear) [88], SVT (Fine-tune) [88], SVFormer-S [89], and SVFormer-B [89] obtain accuracies of 70.5%, 72.2%, 70.7%, 58.6%, 70.3%, 69.0%, 74.8%, 66.2%, 56.5%, 75.1%, 72.3%, 71.5%, 73.7%, 75.9%, 55.6%, 57.8%, 67.2%, 59.7%, and 68.2%, respectively.

For the UCF101 dataset, the proposed DA-CNN+Bi-GRU outperforms all the comparative methods by achieving the best accuracy of 97.6% followed by the runner-up method RTS [90], which attains an accuracy of 96.4%. The multi-task hierarchical clustering method [68] attains the lowest accuracy of 76.3% amongst all the comparative methods, followed by SVFormer-S [89], which achieves the second-lowest accuracy of 79.1% amongst the other considered comparative methods on the UCF101 dataset. The rest of the comparative methods including saliency-aware 3DCNN with LSTM [91], spatiotemporal multiplier networks [92], long-term temporal convolutions [39], OFF [93], TVNet [94], attention cluster [95], CNN with Bi-LSTM [96], Videolstm [97], two-stream convnets [98], mixed 3D-2D convolutional tube [99], TS-LSTM+temporal-inception [82], TSN+TSM [100], STM [101], correlational CNN+LSTM [84], SVT (Linear) [88], SVT (Fine-tune) [88], ConvNet Transformer [102], and SVFormer-B [89] achieve accuracies of 84.0%, 87.0%, 82.4%, 96.0%, 95.4%, 94.6%, 92.8%, 89.2%, 84.9%, 88.9%, 91.1%, 94.3%, 96.2%, 92.8%, 90.8%, 93.7%, 86.1%, and 86.7%, respectively. Finally, for the Kinetics-600 dataset, the MTV-H [103] achieves the best accuracy of 89.6%, followed by the proposed DA-CNN+Bi-GRU method which attains an accuracy of 86.7%, whereas the MTV-B [103] (variant of MTV-H) achieves an accuracy of 84.0%. The GCF-Net [104] and global and local-aware attention [105] methods attain the lowest accuracies of 70.0% and 70.0%, respectively. The rest of the comparative methods including SlowFast [106], Stnet [107], LGD-3D [108], D3D+S3D-G [109], MoviNet [110], MM-ViT [111], Swin-B [112], and Swin-L [112] achieve accuracies of 81.8%, 76.3%, 82.7%, 79.1%, 83.5%, 83.8%, and 85.9%, respectively. Considering the overall comparative analysis, DA-CNN+Bi-GRU obtains performance comparable to the best-performing method on the YouTube action and Kinetics-600 datasets, and greatly dominates the state-of-the-art comparative methods on UCF50, HMDB51, and UCF101 datasets, thus demonstrating the superiority of our proposed method over the existing action-recognition methods.

**Table 6:** Quantitative comparative analysis of our proposed method with the state-of-the-art action-recognition methods for HMDB51 dataset. The best and the runner-up results are highlighted in bold and italic, respectively.

Method	Year	Accuracy (%)
Multi-task hierarchical clustering [68]	2017	51.4
STPP+LSTM [79]	2017	70.5
Optical flow + multi-layer LSTM [47]	2018	72.2
TSN [80]	2018	70.7
IP-LSTM [81]	2019	58.6
Deep autoencoder [70]	2019	70.3
TS-LSTM + temporal-inception [82]	2019	69.0
HATNet [83]	2019	74.8
Correlational CNN + LSTM [84]	2020	66.2
STDAN [67]	2020	56.5
DB-LSTM+SSPF [48]	2021	75.1
DS-GRU [52]	2021	72.3
TCLC [85]	2021	71.5
Evidential deep learning [78]	2021	*77.0*
ViT+LSTM [76]	2021	73.7
Semi-supervised temporal gradient learning [86]	2022	75.9
AdaptFormer [87]	2022	55.6
SVT (Linear) [88]	2022	57.8
SVT (Fine-tune) [88]	2022	67.2
SVFormer-S [89]	2023	59.7
SVFormer-B [89]	2023	68.2
DA-CNN+Bi-GRU (Proposed)	2023	**79.3**

**Table 7:** Quantitative comparative analysis of our proposed method with the state-of-the-art action-recognition methods for UCF101 dataset. The best and the runner-up results are highlighted in bold and italic, respectively.

Method	Year	Accuracy (%)
Multi-task hierarchical clustering [68]	2017	76.3
Saliency-aware 3DCNN with LSTM [91]	2017	84.0
Spatiotemporal multiplier networks [92]	2017	87.0
Long-term temporal convolutions [39]	2017	82.4
RTS [90]	2018	*96.4*
OFF [93]	2018	96.0
TVNet [94]	2018	95.4
Attention cluster [95]	2018	94.6
CNN with Bi-LSTM [96]	2018	92.8
Videolstm [97]	2018	89.2
Two stream convnets [98]	2018	84.9
Mixed 3D-2D convolutional tube [99]	2018	88.9
TS-LSTM + temporal-inception [82]	2019	91.1
TSN+TSM [100]	2019	94.3
STM [101]	2019	96.2
Correlational CNN + LSTM [84]	2020	92.8
SVT (Linear) [88]	2022	90.8
SVT (Fine-tune) [88]	2022	93.7
ConvNet Transformer [102]	2023	86.1
SVFormer-S [89]	2023	79.1
SVFormer-B [89]	2023	86.7
DA-CNN+Bi-GRU (Proposed)	2023	**97.6**

**Table 8:** Quantitative comparative analysis of our proposed method with the state-of-the-art action-recognition methods for Kinetics-600 dataset. The best and the runner-up results are highlighted in bold and italic, respectively.

Method	Year	Accuracy (%)
SlowFast [106]	2019	81.8
Stnet [107]	2019	76.3
LGD-3D [108]	2019	82.7
GCF-Net [104]	2020	70.0
D3D+S3D-G [109]	2020	79.1
MoviNet [110]	2021	83.5
Global and local-aware attention [105]	2021	70.0
MM-ViT [111]	2022	83.5
Swin-B [112]	2022	83.8
Swin-L [112]	2022	85.9
MTV-B [103]	2022	84.0
MTV-H [103]	2022	**89.6**
DA-CNN+Bi-GRU (Proposed)	2023	*86.7*

### 4.5. Action-Recognition Visualization

**Figure 8:**
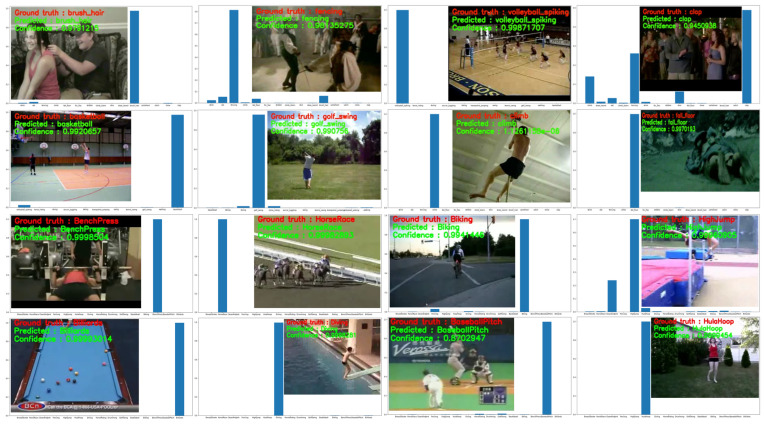
The visual recognition results of our proposed DA-CNN+Bi-GRU framework with predicted classes and their confidence scores for the test videos taken from the YouTube action, UCF50, and HMDB51 datasets.

To validate the recognition efficiency of DA-CNN+Bi-GRU, we tested DA-CNN+Bi-GRU on 15% of test videos taken from each dataset (including YouTube action, UCF50, HMDB51, UCF101, and Kinetics-600 datasets). The prepared test sets are validated for the action-recognition task using our proposed framework and the visual results from the test experiments are depicted in Figure 8. In Figure 8, the representative frames of the predicted action clips are presented along with their ground truths, model predicted actions, and confidence scores over the probability prediction bar graphs for better understanding of readers. It can be perceived from the presented visual results that the DA-CNN+Bi-GRU framework predicts most of the actions including brush hair, volleyball spiking, basketball, climb, fall floor, bench press, horse race, billiards, diving, baseball pitch, and hula hoop with 0.99% probability or 99% confidence. However, for some action classes, such as clap, fencing, golf swing, and high jump, the DA-CNN+Bi-GRU framework also generates non-zero probabilities for wrong action classes; however, these probabilities for wrong action classes are still very low and thus do not affect the prediction of actual action class. Hence, the obtained qualitative visual results verify the effectiveness of our proposed framework for practical use in different vision-based human action-recognition and -monitoring environments.

### 4.6. Runtime Analysis

To analyze the effectiveness and feasibility of the DA-CNN+Bi-GRU framework for practical applications in real-time environments, we estimated the runtime of our method for action-recognition tasks in terms of SPF and FPS with and without using GPU resources. The obtained runtime results are then compared with the state-of-the-art methods. Table 9 presents and compares the runtime of our proposed framework with the running times of the contemporary action-recognition methods. Results in Table 9 demonstrate that the DA-CNN+Bi-GRU framework outperforms the state-of-the-art methods when executing on both GPU and central processing unit (CPU) platforms. Results indicate that our proposed framework attains 0.0036 SPF and 300 FPS while running on GPU, whereas it attains 0.0049 SPF and 250 FPS while running on CPU. Results further show that the second-best execution time results on GPU are achieved by [93], which are 0.0048 SPF and 206 FPS. In Table 9, the best runtime results are indicated with bold text and the runner-up results are indicated with italics. Experimental results indicate that for the SPF metric, the DA-CNN+Bi-GRU framework can provide an improvement of up to 26.1× when running on GPU and an improvement of 87.76× when running on CPU as compared to other contemporary activity-recognition methods. Experimental results further reveal that for the FPS metric, the DA-CNN+Bi-GRU framework can provide an improvement of up to 28.3× when running on GPU and an improvement of up to 166.6× when running on CPU as compared to other contemporary activity-recognition methods.

We also scaled the runtime inference results of the state-of-the-art human action-recognition methods in Table 9 to the hardware specifications used in our framework (i.e., 3.5 GHz CPU and 1607 MHz GPU) to provide a fair comparison of the inference speed. The scaled runtime inference results of the state-of-the-art human action-recognition methods are presented in Table 10. Although scaling does not provide 100% accuracy for processor/GPU runtime because of different instruction set architectures and memory subsystems utilized by different processor/GPU architectures, scaling provides plausible estimates and facilitates relative comparisons [113,114]. From the scaled results in Table 10, it can be seen that the STTP+LSTM [79] method has the best SPF and FPS values of 0.0023 and 423.58, respectively, for the GPU inference. The OFF [93] method has the runner up SPF and FPS of 0.0029 and 331.04, respectively, for the GPU inference, followed by our proposed method which has SPF and FPS of 0.0036 and 300, respectively. Our proposed method thus obtains the third-best SPF and FPS values for the GPU inference. On the other hand, for inference on the CPU, our proposed method delivers the best SPF and FPS values of 0.0049 and 250, respectively, followed by Optical flow + multi-layer LSTM [47], which attains the runner up SPF and FPS values of 0.17 and 3.71, respectively. We note that our method, however, provides a better accuracy on human activity-recognition tasks than the STTP+LSTM [79] method and the OFF [93] method. For the scaled experimental results, it can be observed that for the FPS metric, the DA-CNN+Bi-GRU framework can provide an improvement of 2.82×, on average, when running on GPU and an improvement of up to 94.34×, on average, when running on CPU as compared to other contemporary activity-recognition methods. It is also worth mentioning here that the storage requirement of the DA-CNN+Bi-GRU framework is just 5.4 MB, and thus our framework can be run on resource-constrained IoT and edge devices with very limited memory including today’s smart cameras, Arduino, and Raspberry pi. These runtime and storage requirement results demonstrate that the proposed framework is a suitable candidate for deployment on resource-constrained IoT and edge devices as the proposed framework exhibits better accuracy, lower execution time, and low storage requirements as compared to contemporary activity-recognition methods.

## 5. Conclusions and Future Research Directions

In this work, we proposed a cascaded spatial–temporal discriminative feature-learning framework for human activity recognition in video streams. The proposed method encapsulates the attentional (channel and spatial attention) CNN architecture and bi-directional GRU network as a unified framework for single instance training and efficient spatial temporal modeling of human actions. The attentional CNN architecture comprises channel and spatial attentions, which help retrieve the prominent discriminative features from the object-specific regions, and thus generate high quality saliency-aware feature maps. The bi-directional GRU learns the temporal modeling of long-term human action sequences using two-way gradient learning (i.e., forward and backward pass), which allows the DA-CNN+Bi-GRU framework to utilize the learned knowledge not only from the previous frames but also from the upcoming/next frames. Such bi-directional modeling of human actions greatly helps our method to improve the learning ability while training and the prediction precision while inferencing. To evaluate the efficiency of DA-CNN+Bi-GRU, we conducted extensive experiments on five publicly available human action benchmark datasets. The obtained experimental results are compared with the state-of-the-art methods on five benchmark human action-recognition datasets, including YouTube action, UCF50, HMDB51, UCF101, and Kinetics-600 datasets. Experimental results verify the effectiveness of our method in terms of both model robustness and computational efficiency. Further, we analyzed the runtime performance of our proposed framework in terms of seconds per frame (SPF) and frames per second (FPS) for both CPU and GPU execution environments. The obtained runtime assessment results reveal that our proposed framework can attain an improvement of up to 88× for the SPF metric and up to 167× for the FPS metric as compared to other contemporary action-recognition methods. Additionally, our proposed framework requires a storage of only 5.3 MB, which makes it feasible for deployment on devices with limited memory. Thus, the overall efficiency of our framework in terms of recognition performance (accuracy), low execution time, and low storage requirements, makes DA-CNN+Bi-GRU a strong candidate for real-time IoT and edge applications.

Currently, the DA-CNN+Bi-GRU method only uses the spatial attention (channel and spatial attention) mechanism, which is indeed very effective. However, in the future, we plan to use the temporal attention mechanism together with spatial attention because such hybrid attention has a great potential to improve the human activity-recognition performance.

## Figures and Tables

**Table 1:** Architectural details of our proposed CNN architecture.

Layer	Input Channels	Number of Kernels	Kernel Size	Stride	Padding	Output Channels
Conv 1	3	16	3×3	1	1	16
Conv 2	16	16	3×3	1	1	16
Max pooling						
Channel Attention						
Spatial Attention						
Conv 3	32	32	3×3	1	1	32
Conv 4	32	32	3×3	1	1	32
Max pooling						
Channel Attention						
Spatial Attention						
Conv 5	32	32	3×3	1	1	32
Conv 6	32	32	3×3	1	1	32
Max pooling						
Channel Attention						
Spatial Attention						
Conv 7	32	64	3×3	1	1	64
Conv 8	32	64	3×3	1	1	64
Max pooling						
Channel Attention						
Spatial Attention						
Global Average Pooling						
Flatten						

**Table 2:** Network settings of experimented baseline methods and our proposed framework.

Method	Spatial Block Layers	Temporal Block Layers
CNN+LSTM	8 convolutional	3 LSTM
CNN+Bi-LSTM	8 convolutional	6 LSTM (3 forward and 3 backward)
CNN+GRU	8 convolutional	3 GRU
CNN+Bi-GRU	8 convolutional	6 GRU (3 forward and 3 backward)
DA-CNN+Bi-GRU	12 convolutional (8 convolutional and 4 attentional)	6 GRU (3 forward and 3 backward)

**Table 3:** Quantitative comparative analysis of our proposed framework with other baseline methods. The best and the runner-up results are highlighted in bold and italic, respectively.

Method	Dataset	Accuracy (%)
CNN+LSTM	YouTube action	64.7
CNN+Bi-LSTM	YouTube action	84.2
CNN+GRU	YouTube action	88.5
CNN+Bi-GRU	YouTube action	92.1
CNN (channel attention only)+Bi-GRU	YouTube action	94.2
CNN (spatial attention only)+Bi-GRU	YouTube action	*95.6*
DA-CNN+Bi-GRU (Proposed)	YouTube action	**98.0**
CNN+LSTM	UCF50	76.3
CNN+Bi-LSTM	UCF50	83.3
CNN+GRU	UCF50	87.6
CNN+Bi-GRU	UCF50	93.6
CNN (channel attention only)+Bi-GRU	UCF50	95.1
CNN (spatial attention only)+Bi-GRU	UCF50	*95.7*
DA-CNN+Bi-GRU (Proposed)	UCF50	**98.5**
CNN+LSTM	HMDB51	56.7
CNN+Bi-LSTM	HMDB51	63.2
CNN+GRU	HMDB51	68.0
CNN+Bi-GRU	HMDB51	72.4
CNN (channel attention only)+Bi-GRU	HMDB51	73.9
CNN (spatial attention only)+Bi-GRU	HMDB51	*74.5*
DA-CNN+Bi-GRU (Proposed)	HMDB51	**79.3**
CNN+LSTM	UCF101	83.9
CNN+Bi-LSTM	UCF101	86.8
CNN+GRU	UCF101	90.7
CNN+Bi-GRU	UCF101	94.2
CNN (channel attention only)+Bi-GRU	UCF101	95.1
CNN (spatial attention only)+Bi-GRU	UCF101	*95.8*
DA-CNN+Bi-GRU (Proposed)	UCF101	**97.6**
CNN+LSTM	Kinetics-600	73.2
CNN+Bi-LSTM	Kinetics-600	77.9
CNN+GRU	Kinetics-600	81.5
CNN+Bi-GRU	Kinetics-600	84.3
CNN (channel attention only)+Bi-GRU	Kinetics-600	84.9
CNN (spatial attention only)+Bi-GRU	Kinetics-600	*85.6*
DA-CNN+Bi-GRU (Proposed)	Kinetics-600	**86.7**

**Table 4:** Quantitative comparative analysis of our proposed method with the state-of-the-art action-recognition methods on YouTube action dataset. The best and the runner-up results are highlighted in bold and italic, respectively.

Method	Year	Accuracy (%)
Multi-task hierarchical clustering [68]	2017	89.7
BT-LSTM [69]	2018	85.3
Deep autoencoder [70]	2019	96.2
STDAN [67]	2020	**98.2**
Two-stream attention LSTM [71]	2020	96.9
Weighted entropy-variance-based feature selection [72]	2021	94.5
Dilated CNN+BiLSTM+RB [73]	2021	89.0
DS-GRU [52]	2021	97.1
Local-global features + QSVM [74]	2021	82.6
DA-CNN+Bi-GRU (Proposed)	2023	*98.0*

**Table 5:** Quantitative comparative analysis of our proposed method with the state-of-the-art action-recognition methods for UCF50 dataset. The best and the runner-up results are highlighted in bold and italic, respectively.

Method	Year	Accuracy (%)
Multi-task hierarchical clustering [68]	2017	93.2
Deep autoencoder [70]	2019	96.4
Ensemble model with swarm-based optimization [75]	2021	92.2
DS-GRU [52]	2021	95.2
Local-global features + QSVM [74]	2021	69.4
ViT+LSTM [76]	2021	96.1
(LD-BF) + (LD-DF) [77]	2022	*97.5*
DA-CNN+Bi-GRU (Proposed)	2023	**98.5**

**Table 9:** Runtime analysis of our proposed framework with the state-of-the-art human action-recognition methods (without scaling). The best and the runner-up results are highlighted in bold and italic, respectively.

Method	Seconds per Frame (SPF)		Year	Frames per Second (FPS)	
	GPU	CPU		GPU	CPU
STPP+LSTM [79]	0.0053	-	2017	186.6	-
CNN with Bi-LSTM [96]	0.0570	-	2017	20	-
OFF [93]	*0.0048*	-	2018	*206*	-
Videolstm [97]	0.0940	-	2018	10.6	-
Optical flow + multi-layer LSTM [47]	0.0356	0.18	2018	30	3.5
Deep autoencoder [70]	0.0430	0.43	2019	24	1.5
TSN+TSM [100]	0.0167	-	2019	60	-
IP-LSTM [81]	0.0431	-	2019	23.2	-
STDN [67]	0.0075	-	2020	132	-
DS-GRU [52]	0.0400	-	2021	25	-
MoviNet [110]	0.0833	-	2021	12	-
(LD-BF) + (LD-DF) [77]	0.0670	-	2022	14	-
DA-CNN+Bi-GRU (Proposed)	**0.0036**	**0.0049**	2023	**300**	**250**

**Table 10:** Runtime analysis of our proposed framework with the state-of-the-art human action-recognition methods scaled to our framework’s hardware specifications. The best and the runner-up results are highlighted in bold and italic, respectively.

Method	Seconds per Frame (SPF)		Year	Frames per Second (FPS)	
	GPU	CPU		GPU	CPU
STPP+LSTM [79]	**0.0023**	-	2017	**423.58**	-
CNN with Bi-LSTM [96]	0.0354	-	2017	32.14	-
OFF [93]	*0.0029*	-	2018	*331.04*	-
Videolstm [97]	0.0584	-	2018	17.03	-
Optical flow + multi-layer LSTM [47]	0.0221	*0.17*	2018	48.21	*3.71*
Deep autoencoder [70]	0.0267	0.40	2019	38.56	1.59
TSN+TSM [100]	0.0167	-	2019	60	-
IP-LSTM [81]	0.0268	-	2019	37.28	-
STDN [67]	0.0046	-	2020	212.12	-
DS-GRU [52]	0.0248	-	2021	40.17	-
MoviNet [110]	0.0645	-	2021	15.48	-
(LD-BF) + (LD-DF) [77]	0.0416	-	2022	22.49	-
DA-CNN+Bi-GRU (Proposed)	0.0036	**0.0049**	2023	300	**250**

## Data Availability

The datasets generated during and/or analysed during the current study are available from the corresponding author on reasonable request.

## Abbreviations

CNN	Convolutional neural network
DA-CNN	Dual attention convolutional neural network
CBAM	Convolutional block attention module
IoT	Internet of things
RNN	Recurrent neural network
GRU	Gated recurrent unit
Bi-GRU	Bi-directional gated recurrent unit
SPF	Seconds per frame
FPS	Frames per second
